# Genomic analysis and antimicrobial resistance of Vibrio cholerae isolated during Zambia’s 2023 cholera epidemic

**DOI:** 10.1099/mgen.0.001566

**Published:** 2025-12-02

**Authors:** Harriet Ng'ombe, Charlie C. Luchen, Lia Bote, Mpanga Kasonde, Kunda Musonda, Kapambwe K. Mwape, Dhvani H. Kuntawala, Suwilanji Silwamba, Mwelwa Chibuye, Kennedy Chibesa, Nyuma Mbewe, Samuel Bosomprah, Wesaal Khan, Lenine Liebenberg, Tulio de Oliveira, Eduan Wilkinson, Matthew J. Dorman, Avril Coghlan, Michelo Simuyandi, Roma Chilengi, Caroline Chisenga, Nicholas R. Thomson

**Affiliations:** 1 Research Division, Centre for Infectious Disease Research in Zambia, Lusaka, Zambia; 2 Centre for Epidemic Response and Innovation, Stellenbosch University, Stellenbosch, South Africa; 3 Amsterdam UMC, Department of Global Health, University of Amsterdam, Amsterdam Institute for Global Health and Development, Amsterdam, Netherlands; 4 Parasites and Microbes Programme, Wellcome Sanger Institute, Hinxton, UK; 5 Zambia National Public Health Institute, Stand 1186, Corner of Chaholi & Addis Ababa Roads, Rhodes Park, Lusaka, Zambia; 6 Department of Biostatistics, School of Public Health, University of Ghana, Accra, Ghana; 7 Microbiology Department, Faculty of Science, Stellenbosch University, Stellenbosch, South Africa; 8 Water Institute, Faculty of Science, Stellenbosch University, Stellenbosch, South Africa; 9 School of Mathematical and Statistical Sciences, College of Science and Engineering, University of Galway, University Road, Galway, H91 TK33, Ireland; 10 Department of Microbiology, Moyne Institute of Preventive Medicine, School of Genetics and Microbiology, Trinity College Dublin, Dublin 2, D02 PN40, Ireland

**Keywords:** antimicrobial resistance, epidemic, Oxford Nanopore Technologies, *Vibrio cholerae*, Zambia

## Abstract

**Introduction.** Cholera, caused by *Vibrio cholerae*, remains a priority public health concern, particularly in developing countries. The first cholera outbreak in Zambia was documented in the 1970s, with recurring epidemics reported since then. In 2023, a cholera outbreak affected Zambia, particularly in districts bordering Malawi, Mozambique and the Democratic Republic of Congo, with significant cases reported in these neighbouring countries. This study aims to analyse cholera cases and isolates obtained during the 2023 epidemic, focusing on geographical distribution, genetic relatedness of isolates and their antibiotic resistance profiles.

**Methods.** Stool samples were collected from patients presenting with cholera-like symptoms across three provinces of Zambia. A total of 98 samples were cultured on thiosulphate citrate bile salts sucrose agar, resulting in 32 sequenced *V. cholerae* isolates. Whole-genome sequencing was performed using Oxford Nanopore Technology, and phylogenetic inference was also achieved by the analysis of SNPs. Phenotypic antimicrobial resistance testing was conducted following Clinical and Laboratory Standards Institute guidelines. The genomic data were analysed for virulence factors and antimicrobial resistance profiles.

**Results.** Of the 98 stool samples tested, 38 confirmed cholera cases were identified. A subset of 32 confirmed *V. cholerae* isolates, predominantly from the Eastern Province of Zambia (*n*=21), was selected for whole-genome sequencing. Genomic analysis revealed that all isolates belonged to the seventh pandemic El Tor lineage and the O1 serogroup, with two distinct clades identified corresponding to the 10th (T10) and 15th (T15) transmission events. Geographical analysis indicated a predominance of Ogawa serotypes in Eastern Province and Inaba in Northern Province. The virulence gene analysis confirmed the presence of key cholera toxin genes (*ctxA* and *ctxB*) and intestinal colonization factors. All isolates carried genes or mutations predicted to confer resistance to multiple antibiotics, including decreased susceptibility to ciprofloxacin, recommended for the treatment of cholera by the World Health Organization.

**Conclusion.** The findings highlight the critical need for enhanced surveillance and targeted interventions to mitigate cholera outbreaks in Zambia. The emergence of resistant *V. cholerae* strains necessitates innovative strategies, including improved water sanitation, vaccination efforts and novel therapeutic approaches to combat this enduring public health threat.

**Impact Statement**

This study significantly enhances our understanding of the 2023 cholera outbreak in Zambia by employing whole-genome sequencing to analyse *Vibrio cholerae* isolates. We reveal the genetic diversity of the circulating strains, identifying their relatedness to regional outbreaks and specific transmission lineages (T10 and T15). The identification of different serotypes (Inaba and Ogawa) and transmission sublineages has implications for outbreak dynamics and potential persistence within specific regions. Given frequent cross-border interactions with neighbouring countries experiencing concurrent outbreaks, understanding the movement and evolution of these strains is crucial for effective regional control efforts. Critically, this study shows the urgent need for enhanced surveillance, regionally coordinated strategies and tailored public health responses. By providing actionable insights into cholera outbreaks, this research empowers policymakers and healthcare professionals to develop targeted interventions that can save lives and prevent future outbreaks in Zambia and beyond.

**Outcome**

This study on the 2023 cholera outbreak in Zambia contributes to the field by providing a detailed genomic analysis of *Vibrio cholerae* isolates, enhancing the understanding of the circulating strains’ characteristics. The identification of specific transmission sublineages (T10 and T15) and their geographical distribution offers valuable insights into the outbreak’s dynamics. Our results are of interest and utility to public health professionals. Genotypic analysis revealed that all isolates carried genes predicted to confer resistance to multiple antibiotics, as well as the presence of core resistance genes floR, sul2, strA and strB in most isolates, with regional variations observed. Specifically, these genes were absent in a subset of T10 isolate genomes, specifically in all T10 from the Northern Province. The significance of this output is substantial, as it emphasizes the importance of enhanced surveillance, targeted interventions and the development of novel therapeutic approaches to combat cholera in Zambia and similar settings.

## Data Summary

All whole-genome sequencing data generated in the study are available in the National Center for Biotechnology Information under the accession number PRJNA1228025. Additionally, accession IDs for samples in the study are listed in the supplementary data. All other supporting data for this study, including accession IDs for public genomes data, have been provided through supplementary data files.

## Introduction

Cholera, caused by the bacterium *Vibrio cholerae*, remains a critical public health issue, particularly in developing countries like Zambia. Worldwide, cholera pandemics have been reported consistently since 1817, with the seventh pandemic, which began in 1961, continuing to the present day [1]. The first cholera outbreak in Zambia was documented in the 1970s, with recurring epidemics reported since then [2]. Zambia’s neighbouring countries, including Mozambique [3], Tanzania [4], Zimbabwe [5] and the Democratic Republic of Congo (DRC) [6], regularly experience cholera outbreaks. In 2022–2023, a cholera outbreak was reported in Zambia, specifically in districts bordering Malawi, Mozambique and the DRC, where combined ongoing outbreaks affected over 80,000 cases [7]. Given frequent cross-border interactions, the risk of the outbreak spreading across Zambia cannot be avoided.

Cholera is an acute diarrhoeal disease caused by the ingestion of water or food contaminated with *V. cholerae* and can result in severe dehydration and death if not treated promptly. More than 200 *V*. *cholerae* serogroups have been isolated, but only serogroups O1 and O139 are known to cause epidemic cholera [8]. Analysis of whole-genome sequences from *V. cholerae* isolates across Africa has revealed at least three significant waves of the seventh cholera pandemic, originating from Asia. These waves further branched into 17 distinct introductions of the seventh pandemic El Tor (7PET) lineage into Africa, designated T1–T17 sublineages [9,11]. These sublineages have enabled the tracking of recent outbreaks, clarified regional transmission patterns and revealed potential routes for recent cholera cases. Of the epidemics reported in Zambia, whole-genome sequencing (WGS) of the 2009/2010 isolates revealed they belonged to the T10 sublineage of 7PET, whilst the 2016 and 2017/2018 isolates were associated with 7PET sublineage T13 [12]. Notably, the 2016 and 2017/2018 isolates also formed separate genetic clusters, differing by at least four single-nucleotide variants (SNVs), with the 2017/2018 isolates showing close genetic relationships with isolates from the 2017 outbreak in Tanzania [13]. Combined, this is consistent with multiple introductions of *V. cholerae* into Zambia rather than the persistence of endemic strains and underlines the need for a regional approach to control.

In addition to frequent introductions and regional spread, *V. cholerae* strains are known to have become resistant to common antibiotics, and the risk of multidrug-resistant (MDR) strains is a growing public health concern. Previous studies in Zambia have shown varying levels of antimicrobial resistance (AMR) to different antibiotics, including nalidixic acid, cotrimoxazole, tetracycline, azithromycin and ciprofloxacin [14,15]. Efforts to manage cholera have relied on the use of antibiotics to shorten illness [16]. However, the increasing prevalence of MDR *V. cholerae* isolates highlights the importance of developing alternative strategies, such as improving water, sanitation and hygiene, promoting vaccination and exploring novel therapeutic approaches.

The 2023 cholera outbreak in Zambia highlights the persistent threat posed by *V. cholerae*, particularly in regions with frequent cross-border interactions and inadequate water, sanitation and hygiene infrastructure. The genetic diversity of isolates, coupled with the emergence of MDR strains, emphasizes the urgent need for enhanced genomic surveillance and regionally coordinated interventions. These findings illustrate that combating cholera requires a multifaceted approach integrating innovative therapeutic strategies, targeted vaccination campaigns and sustainable public health initiatives to mitigate outbreaks and safeguard vulnerable populations in Zambia and beyond.

## Methods

### Isolation and identification of *V. cholerae*

Stool samples were collected from patients presenting at cholera treatment centres and hospitals with cholera-like symptoms, particularly rice water diarrhoea, in three different provinces (Northern, Eastern and Luapula) of Zambia from 19 January 2023 to 21 May 2023. Trained healthcare workers collected samples in sterile containers along with associated metadata, including date of collection and patient residence details. Given the samples were collected during an emergency outbreak, unfortunately, some metadata (for example, dehydration status and demographic breakdown) were inconsistently recorded. The samples were collected in sterile containers and transported to the nearest laboratory facility at 4–8 °C and processed immediately. In some remote areas where transport to the laboratory took longer (~3–4 h), samples were first placed in alkaline peptone water as an enrichment medium and then cultured immediately upon arrival at the laboratory to preserve viability and improve isolation success. The samples were cultured on thiosulphate citrate bile sucrose (TCBS) agar, a selective medium for *Vibrio* species, and incubated at 37 °C for 18–24 h. Of the 98 cultured, 87 presented with characteristic yellow colonies on TCBS, indicative of sucrose fermentation. Biochemical tests were used to confirm the identity of *V. cholerae* isolates. These included oxidase, triple sugar iron (TSI) agar, lysine iron agar (LIA) and sulphide indole motility (SIM) tests. All biochemical reagents and media were sourced from HiMedia Laboratories (Mumbai, India). The expected results consistent with *V. cholerae* are positive oxidase test; TSI agar showing alkaline slant and acid butt (K/A) with no gas production; LIA with lysine decarboxylation indicated by purple slant and butt (K/K); and in the SIM test, negative hydrogen sulphide production (no black precipitate), positive indole production (red layer after Kovac’s reagent) and motility demonstrated by diffuse growth away from stab line [17]. These biochemical profiles, along with colony morphology, were used to confirm presumptive *V. cholerae* colonies prior to serotyping with O1 polyvalent antisera (Mast Group, Germany). Colonies with biochemical profiles characteristic of *V. cholerae* were then subjected to serotyping using slide agglutination with polyvalent antisera for *V. cholerae* O1. To differentiate between the Inaba and Ogawa serotypes, monovalent antisera (Mast Group, Germany) were used.

### Culture and phenotypic antibiotic testing

Kirby–Bauer disk diffusion was used for phenotypic antibiotic testing as previously described [14]. This is a standardized and widely used phenotypic method for antimicrobial susceptibility testing due to its simplicity, cost-effectiveness and reliability. It provides reproducible results by measuring the inhibition zone around antibiotic discs under controlled conditions, which correlate well with minimal inhibitory concentration (MIC) values. *V. cholerae* isolates were previously stored in 15% glycerol with brain heart infusion broth (Sigma-Aldrich^®^ Solutions) at −80 °C. Upon recovery of pure isolates from the freezer, the antibiotic testing started by culture on Mueller–Hinton agar (Sigma-Aldrich Solutions) and incubation at 37.0 °C for 18–24 h to ensure optimal growth conditions and bacterial viability. A critical step in the disc diffusion protocol involved the preparation of standardized bacterial suspensions. A *V. cholerae* suspension was prepared to achieve an inoculum density equivalent to 0.5 McFarland standards or 10^6^ c.f.u. ml^−1^ using a spectrophotometer to ensure accuracy. This corresponds approximately to an optical density (OD) at 600 nm of 0.1 to 0.15, which is standard for this bacterial concentration [18]. The 0.5 McFarland standard ensures that bacterial suspensions contain the appropriate cell density for optimal disc diffusion performance. This standardization is essential for ensuring that zone diameters correlate accurately with MIC reference methods and clinical interpretive criteria [19]. Using sterile cotton-wool swab sticks, each test suspension was streaked onto Mueller–Hinton agar plates. The antibiotics used were ampicillin (10 µg), chloramphenicol (30 µg), cotrimoxazole (25 µg), nalidixic acid (30 µg), erythromycin (15 µg), tetracycline (30 µg) and ciprofloxacin (5 µg). The inoculated agar plates were allowed to stand for 10 min and incubated at 37.0 °C for 18–24 h. Clear inhibition zones were measured in millimetre diameters using a ruler and interpreted as susceptible, intermediate or resistant in accordance with the 2015 Clinical and Laboratory Standards Institute (CLSI) guidelines [20]. Quality control was ensured by simultaneously testing reference strain *Escherichia coli* ATCC 25922 (Sigma-Aldrich^®^ Solutions). The quality control strain was tested alongside the *V. cholerae* isolates to ensure the accuracy and reliability of the antibiotic susceptibility testing. The control strain’s inhibition zone diameter values were compared to established CLSI standards, and any deviations prompted re-evaluation of the test run.

### DNA extraction and *V. cholerae* WGS

**Fig. 1.**
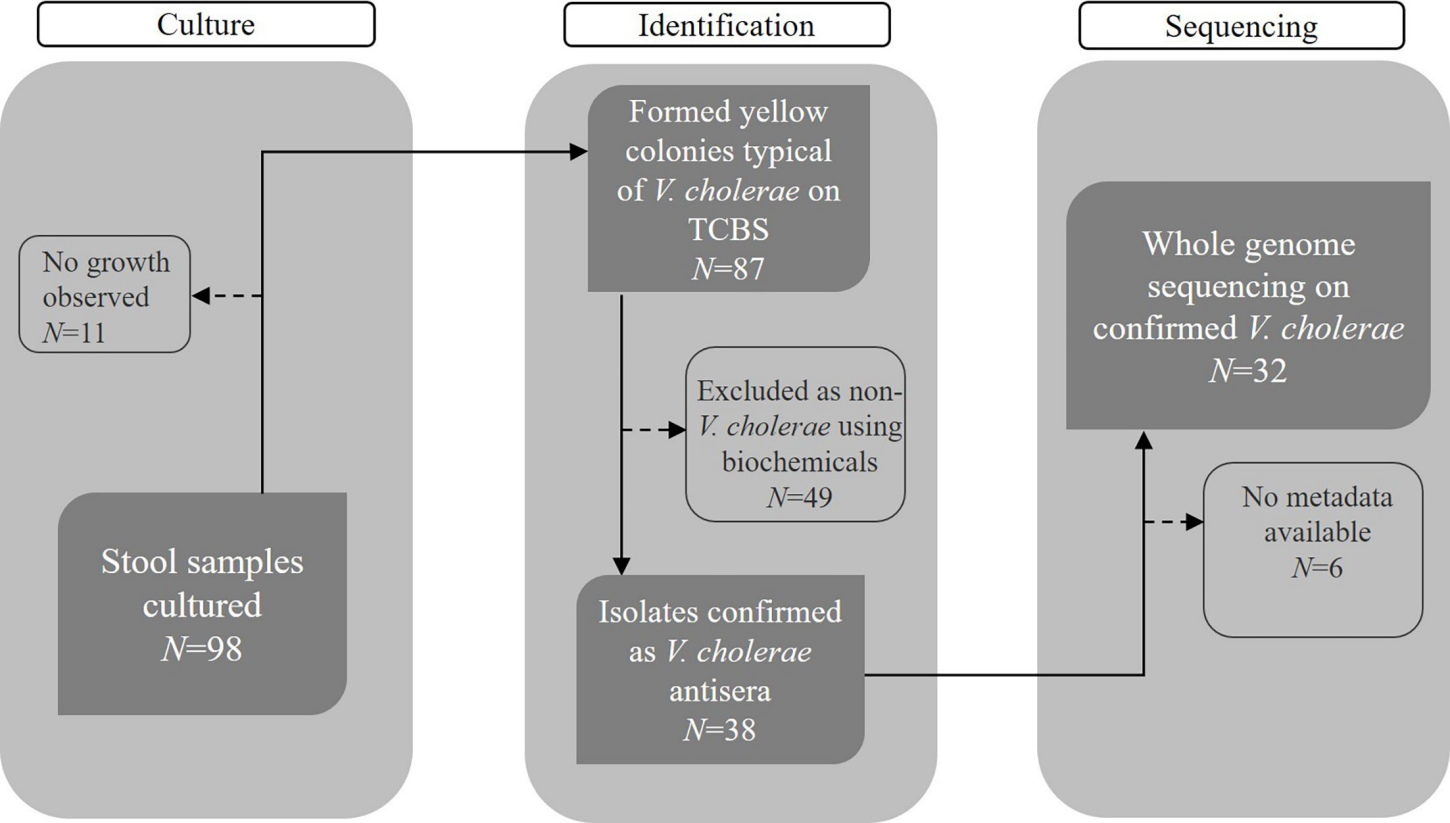
Flow diagram showing the number of samples collected and the final sequencing outcomes. A total of 32 *V*. *cholerae* isolates were sequenced.

Genomic DNA was extracted from the 32 confirmed *V. cholerae* isolates using the Qiagen DNA Mini-Kit protocol as described previously [21]. Briefly, the bacterial cells (grown on Mueller–Hinton) were lysed using Buffer ATL with proteinase K at 56 °C to release nucleic acids. The lysate was then mixed with Buffer AL and ethanol to create optimal binding conditions before being applied to the QIAamp Mini spin column, where DNA binds to the silica membrane whilst contaminants are washed away with Buffers AW1 and AW2. Finally, pure DNA was eluted in Buffer AE, yielding genomic DNA needed for the sequencing. Total nucleic acid from the 32 isolates (Fig. 1) was quantified on the Qubit Fluorometer using the dsDNA High Sensitivity Kit according to instrument instructions. The DNA from confirmed *V. cholerae* isolates was later subjected to WGS on the Oxford Nanopore MK1C platform. Specifically, library preparation followed the Oxford Nanopore Technology (ONT) 1D Native Barcoding Genomic DNA protocol, with modifications [22]. These included DNA repair using NEBNext Companion Module (New England Biolabs), barcode ligation using NEB Blunt/TA Ligase and EXP-NBD104 native barcode kit, pooling of 10–12 DNA samples (total 1 µg DNA) and adapter ligation using NEBNext Quick Ligation Module and SQK-LSK109 ligation sequencing kit. AMPure XP beads were used for sample purification throughout the protocol. MinION sequencing was performed on SpotON Flow Cells (Type R9.4.1) with ~250 ng of library DNA loaded per run. Obtained raw nanopore data were base-called using Guppy v5.0.16 with the ‘superior accuracy’ configuration. Key parameters included barcode demultiplexing for the EXP-NDB104 kit, GPU acceleration using NVIDIA Tesla P100 or V100, barcode trimming, disabled read filtering by median accuracy and disabled telemetry data. Quality control for long reads was performed using pycoQC v2.5.0.3 [23].

### Genome assembly and annotation

*De novo* assembly of the raw reads was performed using the dragonflye pipeline v1.2.1 (https://github.com/rpetit3/dragonflye), with Flye v2.9 [24] as the default assembler. The assemblies were then polished with Medaka v1.4.4 https://github.com/nanoporetech/medaka using the r941_min_sup_g507 model. The final assemblies were assessed for contamination and genome completeness using checkM v.1.2.3 [25]. Subsequent analysis of the assemblies was performed using VibrioWatch, the *V. cholerae* component of Pathogenwatch (https://pathogen.watch/), to obtain species information, lineage assignment, antimicrobial resistance profiles and virulence factors. The Pathogenwatch PAARSNP tool was used for AMR prediction to determine the presence or absence of a resistance gene or variant. Multidrug resistance was defined as resistance to at least three classes of antibiotics or resistance to three or more antibiotics from different drug classes [26].

### Phylogenetic analysis of ONT sequences

Fifty-nine publicly available *V. cholerae* assemblies were used to place the 32 genomes from this study into phylogenetic context. These contextual genomes were selected as they had previously been assigned to named 7PET sublineages associated with transmission waves into Africa (T8–T15) and encompassed spatiotemporal diversity across the continent, collected from 19 countries between 1994 and 2023 [4,9, 27,35] (Data S1, available in the online Supplementary Material 1)

All 91 genomes were shredded into 250 bp single-end pseudo-reads and mapped against the *V. cholerae* reference genome strain N16961 (NZ_LT906614.1 and NZ_LT906615.1) to identify variants, using snippy v4.6.0 (with the --ctgs flag). Due to the inherently high error rate of ONT compared to short-read sequencing, especially in insertions or deletions (indels) and repetitive regions [36,37], we first selected positions only containing SNPs, removing those with indels and complex variants. From these, we extracted sites with SNPs that were present in more than 50% of assemblies and that were not unique to the genomes generated in this study, resulting in 112 selected SNPs.

Based on the 7PET sublineage the contextual genomes have been assigned to in the literature, we selected an additional set of 56 positions with SNPs that were specific to T10, T12, T13 and T15 sublineages. Selected SNPs were validated to be sublineage-specific against a set of genomes belonging to these sublineages that have not been included in this study. The corresponding SNPs were extracted from a total of 168 polymorphic sites, concatenated and aligned with mafft v7.407. Maximum likelihood phylogenetic trees were generated using IQ-TREE v2.3.6 with 1,000 ultrafast bootstraps for the 32 genomes from this study alone and with the 59 contextual genomes included, based on the resulting alignments from selected SNPs.

### Visualization of isolated characteristics

The janitor package version 2.2.0 (https://github.com/sfirke/janitor) was used to clean up and format the datasets. Various R packages such as tidyverse [38], tidyr [39], GGally [40] and Complex Heatmap [41] were utilized for data manipulation and visualization. Phylogenetic trees were visualized using iTOL v7, and the geographic distribution of isolates was plotted using the ‘sf’ package in R v4.4.2.

## Results

### Characteristics of the *V. cholerae* isolates

As shown in the flow diagram (Fig. 1), 98 stool samples were initially cultured, with 11 showing no bacterial growth. Among the 87 samples that formed yellow colonies typical of *V. cholerae* on TCBS agar, 49 were excluded through biochemical testing as non-*V. cholerae*. The remaining 38 isolates were confirmed as O1 *V. cholerae* by serological testing using antisera. Of these confirmed isolates, 32 were selected for WGS, whilst 6 lacked the necessary metadata, e.g. location for sequencing analysis. We then conducted *de novo* assembly and annotation of the 32 sequenced isolates, confirming their identity as *V. cholerae*. All the 32 *V*. *cholerae* genomes had the following characteristics: 2 circular chromosomes, average N50 values of 3.05 Mbp, zero ambiguous bases (non-ATCG) in the assembly and an average G+C content of ~47.5 mol% (Table S2 Supplementary Material 2).

### Geographic distribution and phylogenetic inference of the *V. cholerae* isolates

**Fig. 2.**
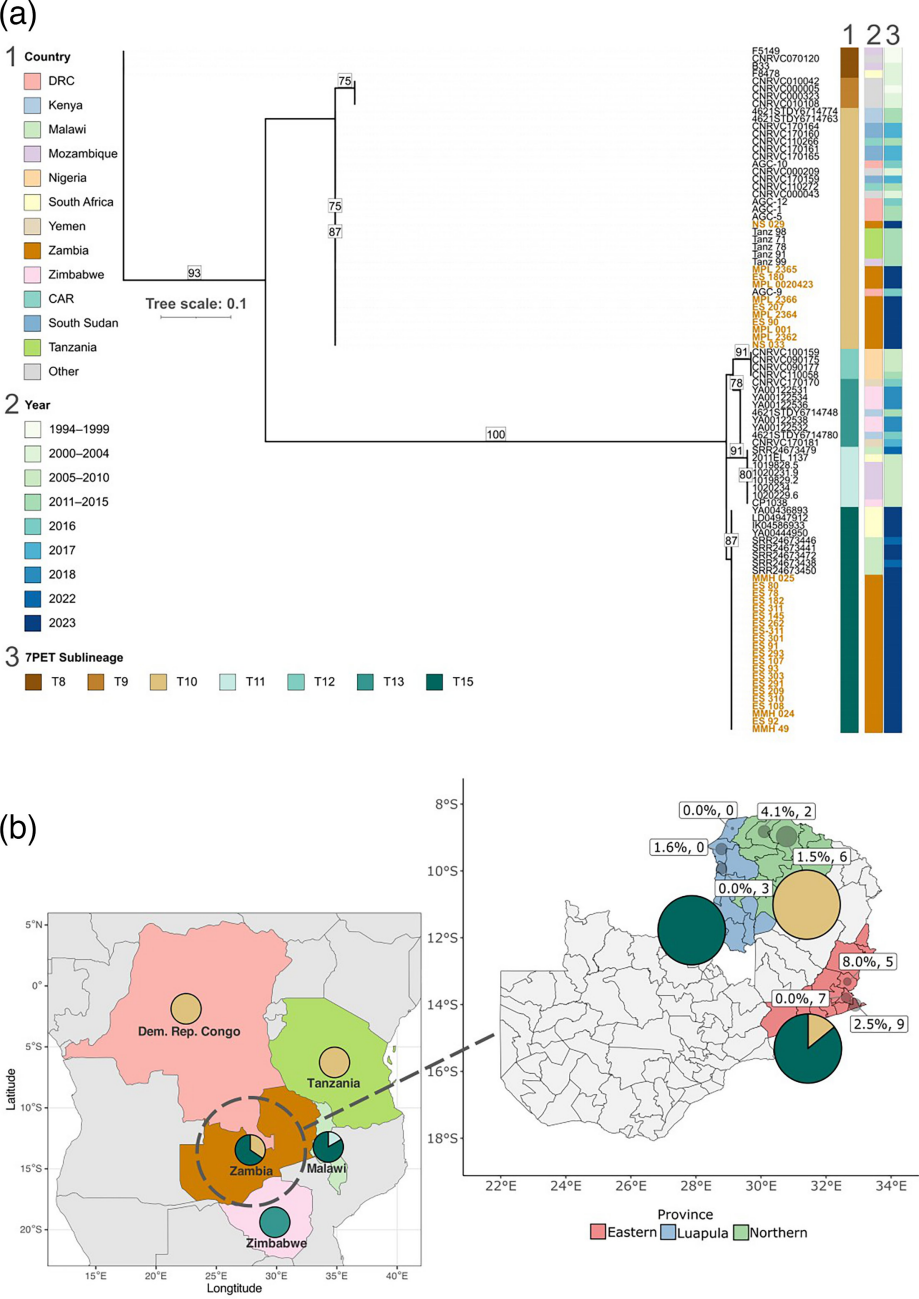
Phylogenetic inference and distribution of the cholera cases in this study. (**a**) Maximum likelihood phylogenetic tree with 1,000 ultrafast bootstraps generated with 168 conserved or lineage-specific polymorphic sites, illustrating the genetic relationships among the 32 *V*. *cholerae* isolates from this study and 59 *V*. *cholerae* isolates from neighbouring countries in Africa, the years and 7PET sublineage. (**b**) Pie charts indicate the proportions of 7PET *V. cholerae* sublineage identified in outbreaks from 2013 to 2023 in Zambia and the neighbouring countries. The inset focuses on these provinces, with text annotations within the inset providing details on case fatality rates (%) and the number of sequenced isolates (*N*) collected from each district. The size of the red dots is proportional to the number of cumulative cases reported in each district.

We were then interested in investigating the geographic distribution of the *V. cholerae* isolates in Zambia and their relationship to outbreaks reported in neighbouring countries (Fig. 2). Of the 32 sequenced isolates, 21 isolates (65.6%) originated from the Eastern Province, specifically from 3 districts: Chipata (7 isolates), Chipangali (5 isolates) and Vubwi (9 isolates). Eight isolates (25%) were from the Northern Province, collected from Nsama (two isolates) and Mpulungu (six isolates), whilst three isolates (9.4%) were from Luapula Province, all from Mwansabombwe district. Epidemiological data showed that Mpulungu reported the highest number of diarrhoea cases (210) with a case fatality rate of 1.5%, whereas Nsama had 73 cases but the highest CFR of 4.1%. Overall, Zambia recorded 552 cholera cases cumulatively from January to July 2023 [42]. Consequently, the samples in this study were collected between 19 January 2023 and 21 May 2023.

Next, we inferred the phylogeny of the 32 isolates from this study and 59 additional genomes from recent outbreaks in the sub-Saharan region [4,5, 9, 27, 31, 35]. All 32 isolates were confirmed as *V. cholerae* belonging to the 7PET lineage. Eleven of these fell within sublineage T10 and clustered with other isolates that have also been previously assigned to the T10 sublineage from countries such as the DRC, Mozambique and Tanzania. The other 21 genomes fell within sublineage T15, clustering with other T15 isolates from Malawi and South Africa. Importantly, the isolates collected in Northern Province all belonged to the T10 sublineage, which was also the dominant sublineage in DRC, Tanzania and other sub-Saharan countries north of Zambia reported during both the earlier years and the same year [43]. In contrast, isolates from the Eastern Province, which borders Malawi, mostly (17/21 isolates) belonged to the T15 sublineage, which was also the most common sublineage circulating in Malawi at the time of this 2023 outbreak [35]. Interestingly, isolates from Luapula (3/3 isolates) belonged to the T15 sublineage, despite being closer to the North, although we only had three genomes sequenced from this region (Fig. 2).

### Characterization of virulence and AMR genes from the Zambian genomes

To understand if there were differences in the virulence potential from the genotypes of the circulating sublineage, we screened the genomes from this study for 23 genes previously linked to *V. cholerae* pathogenicity. Of these, all the virulence genes associated with the 7PET lineage were present, including the cholera toxin, encoded by the *ctxA* and *ctxB* genes, and the toxin-coregulated pilus (TCP), which is responsible for intestinal colonization. Other genes we searched for are involved in functions such as adhesion, regulation, fitness or alternative toxins. The *stn* and *chxA* genes, encoding heat-stable enterotoxin [44] and the cholix toxin [45], respectively, were absent, as is generally the case for 7PET isolates (Data S2 Supplementary Material 2).

We then screened for AMR genes that have been most linked to AMR in *V. cholerae*. The isolates had seven AMR genes: *varG*, *catB9*, *floR*, *sul2*, *strA*, *strB* and *dfrA1*. Based on genome analysis, all the isolates were predicted to be resistant to the following antibiotics: ampicillin, ceftazidime, carbapenems, broad-spectrum cephalosporins, ceftriaxone, furazolidone, nitrofurantoin and trimethoprim. None of the Zambian isolates were predicted to be resistant to azithromycin or tetracycline.

**Fig. 3.**
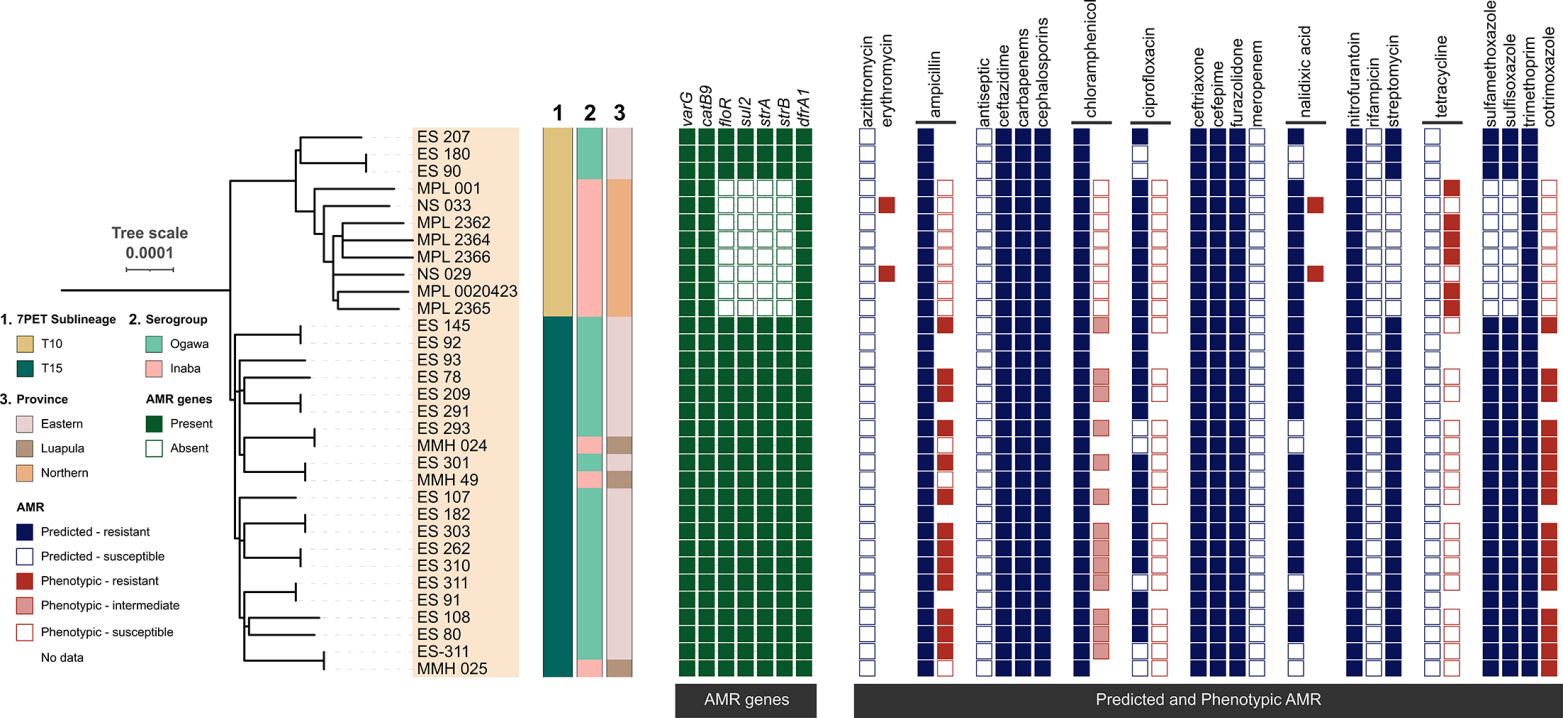
Phylogenetic relationships, genomic context and antimicrobial resistance profiles of *V. cholerae* isolates from Zambia. Left: maximum likelihood phylogenetic tree of 32 *V*. *cholerae* isolates from Zambia annotated by 7PET sublineage (T10, T15), serotype (Ogawa, Inaba) and province of origin. Middle: matrix indicating the presence (green) or absence (white) of key AMR genes or mutations in each isolate. Right: heatmap showing predicted (blue for resistance and white for susceptible) vs. phenotypic (red for resistant or intermediate and white for susceptible) antimicrobial susceptibility profiles for each antibiotic. Columns indicate different antimicrobial agents, while each row corresponds to an individual isolate. The figure illustrates correlations and discrepancies between genotype- and phenotype-based resistance to multiple antibiotics, delineated by sublineages, serotype and geographic distribution.

There were key geographic differences, namely all of the T10 sublineage *V. cholerae* isolates from Northern Province lacked four genes, *floR, sul2, strA* and *strB*, linked to resistance to sulphonamide antimicrobials (including sulfamethoxazole), streptomycin and chloramphenicol. These genes were present in all of the isolates from Eastern Province and Luapula. These genes are located in a discrete insertion within a transmissible sulfamethoxazole–trimethoprim (SXT) element, a mobile genetic element of a class called integrative conjugative elements (ICEs) (Fig. 3).

### Phenotypic AMR profiling of the Zambian genomes

We then performed phenotypic antimicrobial susceptibility testing against seven commonly used antibiotics. Most isolates were susceptible to ampicillin and cotrimoxazole. Tetracycline resistance was only observed in isolates from the Northern Province. Notably, all isolates from the Northern Province, earlier classified as Inaba, were susceptible to ampicillin. In contrast, most from the Eastern Province, earlier classified as Ogawa, were resistant to ampicillin and cotrimoxazole (Fig. 3).

## Discussion

Zambia was one of the first African countries to experience an outbreak linked to the seventh cholera pandemic. First identified in the 1960s in Makassar, Indonesia, the 7PET lineage spread into Africa in the 1970s before reaching the Americas in the 1990s. Since then, there have been more than eleven introductions into Africa, all originating from South Asia [2,9, 46]. Zambia is also surrounded by cholera-endemic countries: between February 2022 and January 2024, Malawi experienced one of its worst outbreaks with over 59,000 cases and a CFR of ~3% [35]. Whilst cholera outbreaks in Malawi typically occur during the rainy season, this particular outbreak extended into the dry season. Similarly, atypical cholera patterns were observed in eastern Zambia, a region not traditionally considered a cholera hotspot [47].

Understanding how *V. cholerae* present in Zambia relates to those in bordering countries is likely critical in breaking the cycle of repeated outbreaks. To achieve the highest possible resolution in characterizing the cholera isolates circulating in Zambia during the 2023 epidemic, we conducted WGS analysis using ONT sequencing. These data revealed that two sublineages of the pandemic 7PET lineage were circulating in Zambia: T10 and T15. Geographically, only T10 isolates were found in the Northern Province, whereas the T15 isolates were predominant in the Eastern Province of Zambia. Looking across Africa, T10 was first seen to have entered Africa in the late 1990s, whereas T15 was first documented in Africa in 2022 [35]. The T10 sublineage is also known to have been responsible for cholera cases over a similar time frame in DRC, Burundi and Tanzania [48]. DRC and Tanzania share land borders with the Northern Province of Zambia, and Burundi is connected to this province through Lake Tanganyika. Our data are consistent with these previous reports highlighting the importance of cross-border transmission and, overall, that the Lake Tanganyika basin is a regional ‘cholera hotspot’ [48]. Unlike previous studies, we showed that the T15 sublineage predominated in the Eastern Province of Zambia and that these isolates were closely related to the T15 sublineage isolates responsible for the protracted unseasonal outbreak in 2022–2023 in Malawi. Malawi also shares a land border with the Eastern Province of Zambia, and, like the Northern Province, there are significant known population movements in both directions across this border. Consistent with this, the T15 isolates are also related to those linked to cases reported in South Africa in 2022–2023, which were postulated to have been imported from Malawi [34]. Whilst T10 and T15 isolates in Zambia showed a strong phylogeographic signal, there were exceptions, with three isolates belonging to T15 seen in Luapula, northern Zambia. It is also important to note that we also documented the T10 sublineage in the samples collected in the Eastern Province, near Malawi, although T10 from the Northern Province was Inaba and lacked the *floR*, *sul2*, *strA* and *strB* but in some cases had tetracycline resistance phenotypes, whilst T10 in the Eastern Province was Ogawa and carried these four genes (Fig. 2), suggesting two genetically and phenotypically different clones of T10. These data also highlight the role of domestic transmission across Zambia.

ONT sequencing with platforms such as the MinION is beneficial, especially in low-resource outbreak settings, as it is portable, cost-effective and requires simpler library preparation than other short- or long-read methods like Illumina or PacBio sequencing. However, sequences generated with the ONT platform are associated with an inherently higher error rate than short-read sequencing, with ONT reads having a sequencing error rate estimated between 5 and 15% compared to a median error rate of between 0.1 and 0.6% across various Illumina short-read sequencing platforms [36,49]. This is especially true for older chemistries like the ONT R 9.4.1 flow cell used in this study [37]. As such, sequencing errors can obscure the infereng of phylogenetic relationships among closely related isolates such as 7PET *V. cholerae*.

Here, we employed a computational method to detect SNPs that were shared between our ONT assemblies and published sequences or that were validated to be phylogenetically restricted and thus less likely to be due to sequencing errors. This allowed us to infer sublineage-level phylogenetic relationships for isolates collected during the 2022–2023 outbreak in Zambia. Whilst this approach may exclude rare and potentially genuine SNPs, it allowed us to generate insight into potential patterns of both domestic and cross-border transmission during this outbreak, which is important for informing disease control.

Looking at microevolutionary events that may characterize the Zambian *V. cholerae* isolate genomes such as primary virulence genes, our analysis showed that all sequenced isolates carried all of those linked to pathogenicity in 7PET, including genes encoding cholera toxin, intestinal colonization factors and transcriptional regulators [50]. CtxAB are the canonical virulence factors that result in the characteristic secretory diarrhoea and are known to be conserved in the 7PET lineage [51]. Further, *toxR* regulates transcription of key virulence factors like CTX, TCP and *toxT* [52,53]. Of the genes we searched for, only *chxA* and *stn* were absent from all genomes. This aligns with other studies that have identified these genes as contributing to virulence in non-7PET lineages, although they remain uncommon [45,54]. It is also worth noting that geographically, there was a predominance of Ogawa serotypes in Eastern Province (mostly from T15) and Inaba in Northern Province (mostly T10), highlighting the complex dynamics of cholera transmission across this region during this epidemic. These findings are evident in the case fatality rates (CFRs) seen during the different outbreaks in Zambia. In 2023, Nsama District in Northern Province recorded the highest CFR at 4.1%. This trend continued in the 2024 outbreak [55], with preliminary data indicating an overall CFR of ~3.2% across affected districts, further stressing the need for immediate and effective public health interventions.

Next, we looked at the genotypic and phenotypic AMR profiles of the isolates studied here. All isolates carried genes or mutations predicted to confer resistance to multiple antibiotics, including ciprofloxacin, recommended for treatment of cholera by the World Health Organization [56]. Importantly, beyond the sporadic loss of individual AMR genes/mutations, our data showed clear regional-specific differences in the presence of core resistance genes *floR*, *sul2*, *strA* and *strB*, all being absent in a subset of T10 isolate genomes, specifically all T10 from the Northern Province. The *floR* gene encodes a membrane efflux pump belonging to the DHA12 family of the major facilitator superfamily and confers high-level resistance to chloramphenicol and its analogues [57]. Genes *strAB* encode aminoglycoside phosphotransferases, proteins conferring resistance to streptomycin [58] through inactivation, and similarly, *sul2* encodes a dihydropteroate synthase that is insensitive to the activity of sulphamethoxazole [59]. In 7PET, these genes are carried on the ICE carrying genes encoding resistance to sulfamethoxazole/trimethoprim named ICE-SXT [60]. This element was first seen in 7PET wave 2 strains isolates in the late 1980s and is now synonymous with wave 2 and wave 3 strains of O1 and O139 7PET *V. cholera*e and broadly distributed across isolates in Africa [46,61].

Within ICE-SXT, the *floR*, *strAB* and *sul2* genes are located on a composite transposon, comprised of multiple insertion events, including the s*trA, strB* and *sul2* gene cassette commonly found together on many plasmids, including RSF1010 [62]. The two genes bordering this cassette, *floR* and *dfrA18,* are located on separate mobile elements. What we see in the Northern Province T10 isolates is the loss of the complete s*trA*, *strB* and *sul2* cassette. This region is stably maintained in all other isolates across both sublineages, including T10 isolates from Eastern Province. Previously, the sequential loss of AMR genes from ICE-SXT, including *floR*, *strAB* and *sul2* [60]*,* was one of the changes linked to the demise of the epidemic *V. cholerae* O139 isolates [61]. *V. cholerae* O139, in the early 1990s, was heralded as representing the eighth cholera pandemic [63]. This loss in resistance genes was thought to have made these O139 isolates uncompetitive compared to the MDR 7PET isolates belonging to waves 2 and 3 [61]. It is noteworthy that the *floR, strAB* and *sul2* genes were also lost from the T13 sublineage isolates in East Africa and Yemen [30] and are absent from all Yemeni 7PET isolates. This deletion appears to have occurred independently in different 7PET sublineages, potentially being selectively neutral in regions where these antibiotics are not commonly used for cholera treatment. Whilst detailed, province-specific antibiotic consumption data for Zambia are limited, variations in healthcare access, prescribing practices and drug availability between the more rural and less developed Northern Province compared to the more urbanized Eastern Province could contribute to differential antibiotic exposure. Whilst most of the differences in antimicrobial resistance profile are explained by independent introductions of different MDR 7PET sublineages, the loss of resistance determinant once in Zambia by T10 in the Northern Province may relate to differential selection due to different treatment practices across Zambia. Similar patterns have been documented in other African settings [14,64], where localized antibiotic consumption strongly influences the distribution and persistence of resistance genes within endemic *V. cholerae* populations. Future studies incorporating comprehensive antimicrobial usage surveys at the regional level in Zambia would greatly enhance understanding of the drivers of resistance gene distribution.

In our study, genotypic predictions of AMR did not consistently align with phenotypic observations. Specifically, for ampicillin and chloramphenicol, which showed the greatest discrepancies, published evidence suggests that *varG,* although generally predictive of a low level of resistance to ampicillin and some cephalosporins [65], is dependent on genetic background to confer complete resistance to beta-lactams [66,67]. Also, whilst we identified the *catB9* gene associated with chloramphenicol resistance, it is known to often be silent in 7PET isolates [9,68], and conversely, only a subset of the Zambian isolates possessed the *floR* gene, the product of which is known to confer a high level of chloramphenicol resistance. Like *floR,* there is also a wealth of other efflux pumps linked to multidrug resistance, which likely explains other discrepancies between the genotypic and phenotypic AMR profiles observed [57,69]. Finally, these discrepancies can also be explained by variability in gene expression and regulation [70].

Overall, the antibiotic resistance profiles observed in our study reflect trends noted in previous outbreaks [14,15]. A study of T15 2022–2023 isolates in Malawi [35] highlighted a notable increase in resistance to tetracycline, a critical antibiotic for cholera treatment, contrasting with the susceptibility observed in most Zambian samples (except for some T10 isolates from Northern Province). Additionally, research from Bangladesh [56] has shown a concerning trend of multidrug-resistant *V. cholerae* strains, with high rates of resistance to ciprofloxacin and azithromycin, raising alarms about treatment efficacy. In comparison, whilst Zambian isolates exhibited resistance to various antibiotics and carried mutations predicted to confer intermediate resistance to ciprofloxacin (Fig. 2), the majority of samples remained phenotypically susceptible to ciprofloxacin and tetracycline.

The findings of this study emphasize the critical need for enhanced public health interventions in Zambia to address the cholera threat1. To reduce endemic spread within Zambia, better testing and prioritization of enhancing water, sanitation and hygiene infrastructure, especially in areas identified as high-risk, are needed. Community education and engagement are essential to promote safe hygiene practices and reduce the risk of cholera transmission. Additionally, integrating genomic surveillance into routine public health monitoring can provide valuable insights into the evolving nature of *V. cholerae* and inform targeted interventions. Given the portability and cost-effectiveness of ONT sequencing, particularly platforms such as the MinION, there is a strong potential to integrate real-time genomic surveillance into national and regional cholera control programmes in Zambia and neighbouring countries. This could enable near real-time outbreak monitoring, rapid identification of transmission chains and early detection of antimicrobial resistance variants, thereby enhancing public health response capabilities. Nevertheless, what is also extremely clear is that cholera outbreaks in Zambia are, in fact, part of much larger multi-country events that cover an ‘epidemiological region’ spanning significant portions of East and Central Africa. Hence, the solution for cholera control in Zambia and bordering countries is to be part of a coordinated regional activity that aims to share intelligence and data that may, through targeted interventions in one country, influence disease burdens and outcomes in another. Ultimately, this is likely to be the only way to control disease in all countries around the Lake Tanganyika basin.

In conclusion, the cholera outbreak in Zambia from January to July 2023 highlights the urgent need for comprehensive coordinated multicountry public health strategies to combat this disease. The genetic diversity, antibiotic resistance and virulence profiles of the circulating *V. cholerae* strains emphasize the challenges involved in managing cholera outbreaks. Ongoing research and surveillance are critical to understanding the dynamics of cholera transmission and resistance, ultimately guiding effective prevention and control measures to protect public health in Zambia and the surrounding regions.

## Supplementary material

10.1099/mgen.0.001566 Supplementary Material 1.

10.1099/mgen.0.001566 Supplementary Material 2. 1

## Abbreviations

AMR: antimicrobial resistance
CFRs: case fatality rates
CLSI: Clinical and Laboratory Standards Institute
DRC: Democratic Republic of Congo
ICEs: integrative conjugative elements
LIA: lysine iron agar
MDR: multidrug-resistant
MIC: minimal inhibitory concentration
ONT: Oxford Nanopore Technology
7PET: seventh pandemic El Tor
SIM: sulphide indole motility
SNVs: single-nucleotide variants
SXT: sulfamethoxazole–trimethoprim
TCBS: thiosulphate citrate bile sucrose
TCP: toxin-coregulated pilus
TSI: triple sugar iron
WGS: whole-genome sequencing
