# Supplementary material for: Genomic analysis and antimicrobial resistance of Vibrio cholerae isolated during Zambia’s 2023 cholera epidemic
Source: Microb Genom. 2025 Dec 2;11(12):001566. doi: 10.1099/mgen.0.001566 (PMC12671739; doi:10.1099/mgen.0.001566)
Supplement: Supplementary Material 2. [file mgen-11-01566-s002.pdf]

Figure S2

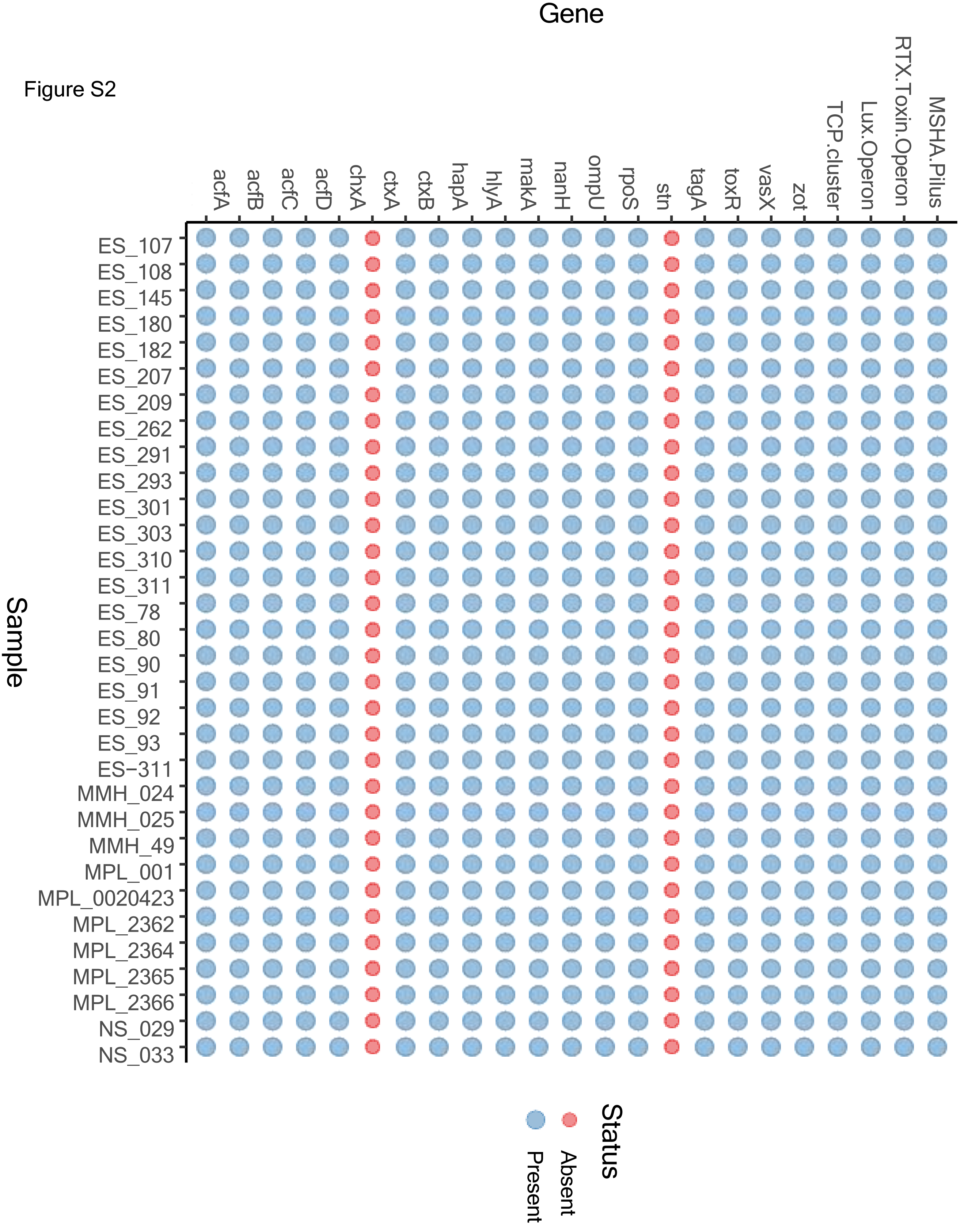
Gene
acfA
acfB
acfC
acfD
chxA
ctxA
ctxB
hapA
hlyA
makA
nanH
ompU
rpoS
stn
tagA
toxR
vasX
zot
TCP.cluster
Lux.Operon
RTX.Toxin.Operon
MSHA.Pilus
Sample
ES_107
ES_108
ES_145
ES_180
ES_182
ES_207
ES_209
ES_262
ES_291
ES_293
ES_301
ES_303
ES_310
ES_311
ES_78
ES_80
ES_90
ES_91
ES_92
ES_93
ES−311
MMH_024
MMH_025
MMH_49
MPL_001
MPL_0020423
MPL_2362
MPL_2364
MPL_2365
MPL_2366
NS_029
NS_033
Status
Absent
Present

Data S2 Phylogenetic analysis

The 32 assemblies generated in this study and a collection of 59 publicly available *V. cholerae* genomes from Africa, including those that have been previously assigned to transmission waves T8, T9, T10, T11, T12, T13, and T15, were shredded into 250 bp single-end pseudo-reads using snippy v4.6.0 with the --ctgs flag. These were then mapped against *V. cholerae* N16961 (NZ_LT906614.1, NZ_LT906615.1) to identify variants. In order to address the sequencing errors specifically associated with the ONT assemblies from this study, polymorphic sites were only included if they were found in more than 50% of assemblies and were not unique to the genomes from this study. An additional set of lineage-specific SNPs were also included, and a concatenated alignment was generated for these 91 assemblies from a total of 168 polymorphic sites. These were aligned with mafft v7.407 and used as input to build a maximum-likelihood phylogenetic tree using IQTREE v2.3.6 with 1000 ultrafast bootstraps. Trees were visualised using iTOL v7 and the geographic distribution of these isolates was plotted using the ‘sf’ package in R v4.4.2.

---

1. Break assemblies into 250bp pseudo-reads and map to N16961 with snippy
2. Filter out SNPs (only, not indels/ complex variants) from each genome and save into individual `snps_only_filt.tab` files
3. Make a list of all loci with SNPs, combine into `snps_pos_all`, with chr and locus ID separated by "_"
4. Count occurrences of loci with SNPs and filter out those that are present in more than just the 32 Zambia ONT assembles, with `for i in $(cat snippy/snps_pos_all);  do python count_item_in_files.py ${i} snippy/out.*/snps_only_filt_pos ; done | awk '$2 >= 33 {print $1}' | sed 's/_/\t/' >> snippy/snps_in_n33_up #found 112 SNPs; also tried for SNPs present in ≥80% of genomes, but not granular enough for tree`
5. Get corresponding SNPs (from snippy .tab output) across these loci in all assemblies with `for i in $(cat assembly_ids); do while IFS=$'\t' read -r col1 col2 ; do grep -P "${col1}\t${col2}" snippy/out.${i}/snps_only_filt.tab; done < snippy/snps_in_n33_up >> snippy/conserved_snps_per_genome/conserved_snps_n33_up.${i}.tab ; done`
6. Concatenate all SNPs across these sites in all genomes with `for i in $(cat assembly_ids); do less snippy/conserved_snps_per_genome/conserved_snps_n33_up.${i}.tab | awk '{print $5}' | tr '\n' ' ' | sed 's/[[:blank:]]//g' >> snippy/conserved_snps_per_genome/snps_${i}.fasta ; done`
7. Add FASTA header and line break with `for i in $(cat assembly_ids); do sed "1s/^/>${i}\n/; \$a\\" snippy/conserved_snps_per_genome/snps_${i}.fasta ; done > snps_multi_n33_up.fasta`
8. Generate MSA with MAFFT

9. T12, T13, and T15 look the same on tree, so I found SNPs present in T12 but not T13, and T13 but not T15, and T15 but not T13, then re-ran everything from step 5
10. Added 15 SNPs that were T10-specific → 168 in total
11.

Custom <count_item_in_files.py> script:

```
import argparse
import re
def count_item_in_files(item, file_paths):
    """
    Counts how many times the item appears across all provided text
files.

    :param item: The item (word, phrase, or character) to count.
    :param file_paths: List of file paths containing text data.
    :return: The total count of the item.
    """
    total_count = 0
    for file_path in file_paths:
        try:
            with open(file_path, 'r', encoding='utf-8') as file:
                for line in file:
                    total_count +=
len(re.findall(rf'\b{re.escape(item)}\b', line))
        except Exception as e:
            print(f"Error reading file {file_path}: {e}")
    return total_count

def main():
    # Set up argument parser
    parser = argparse.ArgumentParser(description="Count occurrences of
an item across multiple tex>
    parser.add_argument("item", help="The item (word, phrase, or
character) to count.")
    parser.add_argument("files", nargs="+", help="Paths to text
files.")
    args = parser.parse_args()

    # Count occurrences of the item across files
    count = count_item_in_files(args.item, args.files)
    print(f"{args.item}\t{count}")

if __name___== "__main__":
    main()
```

Table S2: Genomic profiles of the assemblies according to VibrioWatch

SAMPLE	GENOME LENGTH	N50	NUMBER OF CHROMOSOMES	NON-ATCG	PERCENT GC CONTENT
**ES_180**	4100174	3059323	2	0	47.5
**ES_90**	4100174	3059323	2	0	47.5
**ES_207**	4100190	3059342	2	0	47.5
**ES_145**	4097584	3048501	2	0	47.5
**ES_92**	4097584	3048501	2	0	47.5
**ES_293**	4102807	3048401	2	0	47.5
**MMH_024**	4102807	3048401	2	0	47.5
**ES-311**	4097582	3048472	2	0	47.5
**MMH_025**	4097582	3048472	2	0	47.5
**ES_291**	4097574	3048458	2	0	47.5
**ES_209**	4097574	3048458	2	0	47.5
**MMH_49**	4097537	3048466	2	0	47.5
**ES_301**	4097537	3048466	2	0	47.5
**ES_182**	4097497	3048401	2	0	47.5
**ES_303**	4097497	3048401	2	0	47.5
**ES_262**	4097620	3048478	2	0	47.5
**ES_310**	4097620	3048478	2	0	47.5
**ES_91**	4097591	3048459	2	0	47.5
**ES_311**	4097591	3048459	2	0	47.5
**MPL_2362**	4088915	3048099	2	0	47.4
**MPL_2365**	4088904	3048039	2	0	47.4
**MPL_2364**	4088886	3048062	2	0	47.4
**MPL_2366**	4088975	3048098	2	0	47.4
**MPL_0020423**	4088888	3048060	2	0	47.4
**NS_033**	4088948	3048074	2	0	47.4
**NS_029**	4088496	3047792	2	0	47.4

**NS_080**	4097506	3048418	2	0	47.5
**ES_107**	4097497	3048390	2	0	47.5
**ES_108**	4088663	3047821	2	0	47.4
**MPL_001**	4097463	3048394	2	0	47.5
**NS_025**	4097537	3048415	2	0	47.5
**NS_010**	4097594	3048458	2	0	47.5

Genomic profiles were analyzed using Vibriowatch, a tool for studying *V. cholerae* genomic data. The table presents the genome length, N50, number of chromosomes, non-ATCG content, and percent GC content for each sample. For more information on Vibriowatch, see *https://vibriowatch.readthedocs.io/*
